# CX3CR1 in multiple sclerosis

**DOI:** 10.18632/oncotarget.4650

**Published:** 2015-06-25

**Authors:** Serge Rivest

**Affiliations:** Department of Molecular Medicine, Faculty of Medicine, Neuroscience Laboratory, CHU de Québec Research Center, Laval University, Quebec, Canada

**Keywords:** Immunology and Microbiology Section, Immune response, Immunity

In a recent paper published in J. Ex. Med, we provided evidence that the knockout of CX3CR1 blocked the clearance of myelin debris by microglia, which greatly affected the integrity of axons and myelin sheaths, preventing proper remyelination. These results highlight the crucial role played by CX3CR1 in myelin removal and show that there can be no efficient remyelination following a primary demyelinating insult if myelin clearance by microglia is impaired. We also demonstrated a marked CCR2-dependent infiltration of bone marrow-derived cells in demyelinating areas but these cells do not impact demyelination and remyelination.

Multiple Sclerosis (MS) is a chronic inflammatory disorder of the central nervous system (CNS) associated with prominent demyelination, which impairs the conduction of signals along axons. While the therapeutic control of immune processes has proven efficient to limit the relapsing-remitting form of MS, the current immunomodulatory treatments have failed to prevent patients from entering a progressive course of the disease or to limit disease progression in this phase. Remyelination is the natural regenerative mechanism to counter demyelination, but the reasons why remyelination fails or is incomplete during MS are not completely understood.

Myelin removal is a critical step in the remyelination process (Kotter *et al*., 2006). Cells of the mononuclear phagocytic system, including monocyte-derived macrophages (MDM) and microglia, are actively implicated in the clearance of myelin debris. Microglia are resident macrophages of the CNS that originate from progenitors in the embryonic yolk sac populating the brain during early embryogenesis. Monocytes are macrophage precursors in the circulation and derive from bone marrow progenitors. While monocytes do not migrate into the CNS under normal conditions, we and others have shown the specific infiltration of MDMs under pathological conditions (Lampron *et al*., 2013, 2012). Microglia and macrophages are regarded as detrimental in MS and experimental autoimmune encephalomyelitis (EAE), through their roles in autoimmunity such as antigen presentation and pro-inflammatory cytokine production. However, these noxious roles might mask other beneficial properties. During demyelination, microglia exert a phenotype associated with phagocytosis and the recruitment of oligodendrocyte precursor cells (OPC). While recent work unraveled differences between the roles played by blood-borne macrophages and microglia during autoimmune-mediated demyelination (Yamasaki *et al*., 2014), their respective functions in the process of primary de- and remyelination of the brain were not completely understood until recently.

In a recent study published in J. Ex. Med (Lampron *et al*., 2015), we used the cuprizone model to study the differential roles played by monocytes versus microglia during demyelination and remyelination. Cuprizone is a copper-chelating toxin that induces apoptosis of oligodendrocytes (OD) when added to standard rodent chow. After removal of cuprizone from the diet, complete remyelination of affected sites occurs over the course of 1 to 3 weeks. While significant recruitment of monocytes from the circulation to the demyelinating sites occurs during the demyelination phase, these cells do not impact de- or re-myelination. On the other hand, knocking out CX3CR1 severely impeded phagocytosis of myelin by microglia, leading to the persistence of myelin debris throughout the white matter of cuprizone fed mice. This resulted in inefficient axonal remyelination characterized with aberrant myelin patterns, thus demonstrating the critical role of CX3CR1 in promoting microglial clearance of degenerate myelin and its importance for an efficient remyelination of demyelinating sites.

Microglia are actively implicated in myelin removal during demyelination. The CX3CL1/CX3CR1 axis is crucial in the maintenance of microglia under a resting state because of the constant crosstalk with neurons. Our data suggest that the resistance of CX3CR1−/− mice to demyelination does not result from a resistance to cuprizone treatment, but rather to a defect in myelin removal by CX3CR1−/− mice. These mice displayed both a defect in microglial migration to the corpus callosum and a defect in phagocytosis of myelin debris while inflammatory responses were similar to their wild-type (WT) littermates. Electron microscopy revealed an almost complete absence of phagocytic inclusions in CX3CR1−/− microglia, unlike WT microglia that were gorged with such inclusions. This suggested that a poor phagocytosis of microglial cells is the primary shortcoming responsible for impaired clearance of myelin debris in CX3CR1−/− mice.

Immunomodulators have been very efficient in the control of acute demyelinating events in relapsing-remitting courses of MS. However, these molecules have largely been ineffective in alleviating chronic demyelination in progressive forms of the disease, suggesting a primary neurodegenerative course (Stys *et al*., 2012). As autoimmunity does not seem to play a significant role in progressive forms of MS, a balance between limiting demyelination and boosting remyelination of affected sites must be reached for long-term therapeutic support of these patients (Fox *et al*., 2012). However, the results reported in this study propose important new concepts to take into consideration: there can be no efficient remyelination if microglia are unable to clear degenerate myelin from affected axons. As such, an optimal treatment strategy could consist in boosting microglial phagocytic processes while limiting inflammatory responses, combined with agents targeting OD physiology. Monocytes and microglia are easily targetable, even with compounds that do not cross the BBB, making them prime candidates for novel therapeutic options in progressive forms of demyelinating diseases. The use of macrophage-colony stimulating factor (M-CSF) may be particularly powerful in that regard and we are currently testing it in this mouse model of primary progressive MS.

